# Wild Food Plants and Trends in Their Use: From Knowledge and Perceptions to Drivers of Change in West Sumatra, Indonesia

**DOI:** 10.3390/foods9091240

**Published:** 2020-09-04

**Authors:** Lukas Pawera, Ali Khomsan, Ervizal A.M. Zuhud, Danny Hunter, Amy Ickowitz, Zbynek Polesny

**Affiliations:** 1 Faculty of Tropical AgriSciences, Czech University of Life Sciences Prague, Kamýcká 129, 16500 Praha-Suchdol, Czech Republic; paweralukas@gmail.com; 2 The Indigenous Partnership for Agrobiodiversity and Food Sovereignty, c/o The Alliance of Bioversity International and CIAT, Via dei Tre Denari 472, 00054 Rome, Italy; 3 Department of Community Nutrition, Faculty of Human Ecology, IPB University, Bogor 16680, Indonesia; erlangga259@yahoo.com; 4 Department of Forest Resources Conservation and Ecotourism, Faculty of Forestry, IPB University, Bogor 16680, Indonesia; eamzuhud@apps.ipb.ac.id; 5 Alliance of Bioversity International and CIAT, via dei Tre Denari 472/a, 00054 Rome, Italy; d.hunter@cgiar.org; 6 Center for International Forestry Research, Bogor 16115, Indonesia; a.ickowitz@cgiar.org

**Keywords:** wild edible plants, indigenous foods, agrobiodiversity, nutrition and diets, food systems, food environment, local knowledge, ethnobotany

## Abstract

Wild food plants (WFPs) are often highly nutritious but under-consumed at the same time. This study aimed to document the diversity of WFPs, and assess perceptions, attitudes, and drivers of change in their consumption among Minangkabau and Mandailing women farmers in West Sumatra. We applied a mixed-method approach consisting of interviews with 200 women and focus group discussions with 68 participants. The study documented 106 WFPs (85 species), and Minangkabau were found to steward richer traditional knowledge than Mandailing. Although both communities perceived WFPs positively, consumption has declined over the last generation. The main reasons perceived by respondents were due to the decreased availability of WFPs and changes in lifestyle. The contemporary barriers to consuming WFPs were low availability, time constraints, and a limited knowledge of their nutritional value. The key motivations for their use were that they are free and “unpolluted” natural foods. The main drivers of change were socio-economic factors and changes in agriculture and markets. However, the persistence of a strong culture appears to slow dietary changes. The communities, government and NGOs should work together to optimize the use of this food biodiversity in a sustainable way. This integrated approach could improve nutrition while conserving biological and cultural diversity.

## 1. Introduction

Although the current global food system is believed to be capable of providing enough calories for the world, there are still around two billion people who experience hunger or do not have access to a nutritious diet [1]. An increasing number of countries experience the double burden of malnutrition, where undernutrition coexists with overweight, obesity and other diet-related diseases [2]. Recent studies have demonstrated that food systems are failing to deliver a healthy diet and are inequitable and environmentally unsustainable [3,4]. Global trade and markets play an omnipresent role in influencing human dietary and lifestyle habits, and among Indigenous and vulnerable communities, tend to increase the consumption of highly processed foods of poor nutrient value [5]. For these many reasons, traditional landscapes, cultures and foodways are increasingly homogenized, and many communities are undergoing a nutritional transition negatively affecting their health [6,7].

Wild food plants (WFPs) have been part of diets and traditional food systems throughout human history, providing important nutrients and bioactive compounds. Ancestral and contemporary traditional diets are known to offer valuable health benefits [8]. There are also suggestions that humans and their genome are adapted to the diet and environment from past times and that contemporary diets and lifestyles are not optimal for the human genome [9]. The Western dietary pattern is characterized by a high consumption of ultra-processed foods, which also seems to push the human gut microbiome to produce negative health outcomes and inflammation [10]. WFPs are traditional foods that tend to be richer in micronutrients than cultivated crops [11,12]. This offers the potential for alleviating micronutrient deficiencies in some contexts such as among rural and Indigenous communities [13,14]. WFPs also represent bioactive functional foods that could contribute to healthy diets and immunity to a variety of illnesses [15,16]. Among Indigenous communities, a higher use of wild foods has been linked with greater food security [17]. WFPs are embedded in traditional food knowledge, which represents an integral part of local and sovereign food systems [18].

Despite their potential benefits, WFPs have been overlooked and excluded from most formal education, policies and research or development programs. The barriers to a greater use of WFPs were reviewed by [19], with the main ones being a lack of information, statistics, market infrastructure, research and policies. Moreover, food and agriculture sectors have neglected wild species in favor of cash crops and starchy staples [20]. In terms of research, documentation of WFPs is challenging and numerous assessments and “production diversity” studies fail to capture them as they are uncultivated and stewarded in the social memory of communities. Quantifying their contribution to diets is also limited by a severe lack of food composition data [13]. In addition, their free availability in nature has resulted in low economic valuation, which further reduces their visibility and promotion despite their nutritional, health, social and ecological benefits [21].

Currently, numerous drivers are accelerating the decline in biodiversity and the use of WFPs, such as changes in land-use, climate change, agriculture intensification, overharvesting, socio-economic change, expansion of markets and the loss of local knowledge [20,22].

In Indonesia, one of the most bioculturally diverse countries in the world, foods and diets vary along with geographical, socio-economic and cultural diversity [23]. Food in Indonesia has a high socio-cultural value [24]. Despite its gastronomical richness and significant economic growth, malnutrition in Indonesia remains a major problem [25]. In 2018, the national prevalence of stunted children under 5 years was 29.9%, and anemia reached 48.9% of pregnant women [26]. The problem is multi-faceted [27], but in terms of diet, the main issue appears to be an extreme dependence on rice and a low intake of nutritious foods such as fruits and vegetables [23]. According to the same authors, Indonesians should substitute refined rice with a wider variety of staple foods; increase intake of traditional fruits and vegetables; increase consumption of proteins and fats, especially among those who are undernourished; and curb processed foods rich in added sugars and oils. As shown by the PROSEA (Plant Resources of South East Asia: http://proseanet.org/prosea/) series, Indonesia is rich in wild and cultivated foods, but the country is losing its forests and biodiversity at a tremendous rate [28] and little is known about changes in WFPs.

In West Sumatra, local communities maintain a relatively diverse diet with a prevalence of traditional foods [29,30]. But the quality of the diet was found to be rather low, mainly due to a monotonous diet and a high intake of saturated fatty acids [31]. There is also a high incidence of diet-related problems, such as coronary heart disease and anemia [30]. A previous survey showed that rural West Sumatra is rich in wild and cultivated food plants which could improve the diet [29]. But many of these plant foods are under-consumed. Our understanding of the barriers, motivations and reasons for changes in the use of these foods is limited in Indonesia, resulting in a lack of action to address this issue. Here we present a case study in West Sumatra as an attempt to understand the reasons behind the change in WFP use in the context of a traditional food system.

The objectives of this study were: (1) to document and assess the local knowledge on WFPs of the Minangkabau and Mandailing communities; (2) to understand the perceptions and attitudes on WFPs; and (3) to explain the reasons for changes in the use of WFPs along with the drivers of these changes.

## 2. Materials and Methods

### 2.1. Study Area

**Figure 1:**
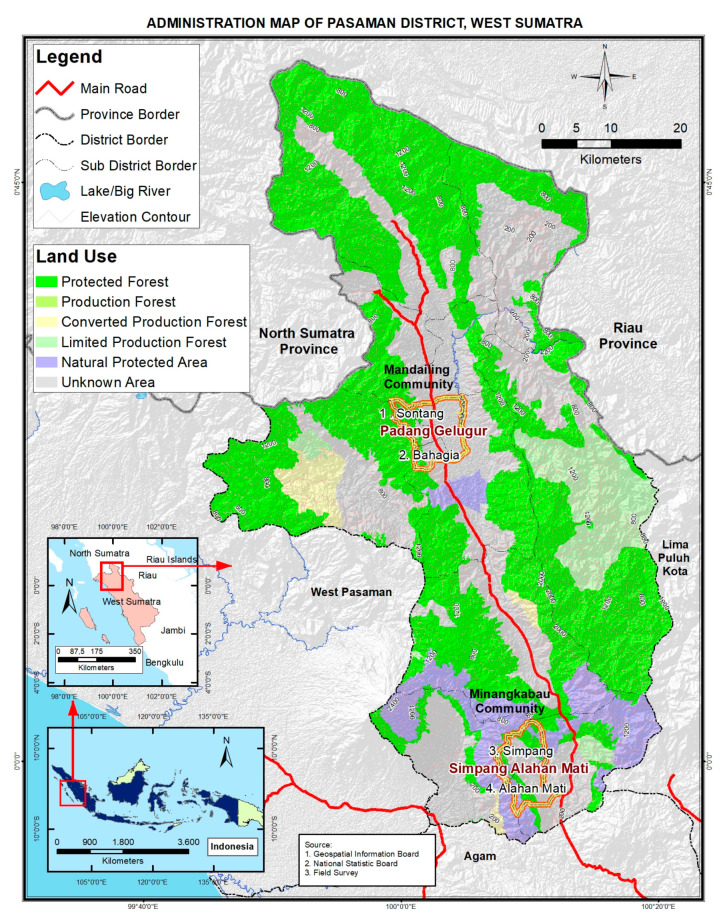
Map of the study area.

West Sumatra province lies in the range of the Bukit Barisan Mountains, with the western part aligned with the Indian Ocean. The province has an area size of about 42,297.30 km^2^ divided into 12 regencies [32]. The region falls in the tropical wet climate zone with rainy and dry seasons. The montane rainforests receive rainfall, which averages more than 2500 mm/year [33]. The area is rich in plant and animal biodiversity, with iconic species being tigers, orangutans, gibbons, or the Rafflesia plant, Andalas tree (*Morus macroura*), or endemic orchids. Tropical forests that in the past dominated the area are restricted to mostly protected areas and only a few customary forests, “hutan adat”. The province is dominated by a mosaic landscape which has been maintained by traditional land management based on the strong relationship between the Minangkabau people and their land. The core of local land-use systems is based on the cultivation of wet rice and agroforestry systems dominated by trees [34]. Rice fields are situated close to settlements as they need intensive care and water management. Forestland and mixed agroforestry systems are situated in hilly areas where the lower soil fertility and the more frequent erosion is more suitable for growing trees than annual plants [35]. The most important lowland crops are rice, coconut and chili, while hill slopes are dominated by cocoa, rubber, coffee, durian, cinnamon, clove tree, and numerous other fruit or multipurpose trees. Our study area is located in the Pasaman regency, which is isolated, landlocked and has a high cover of forests (Figure 1). The selected regency has the highest rate of stunted children in the province, reaching 41% [36].

### 2.2. Study Communities

From a cultural perspective, the region is dominated by the Minangkabau ethnic group and to a lesser extent by the Mandailing ethnic group, which is more populous in North Sumatra [37]. The study area was located at a cultural crossroad in the north of West Sumatra and included both ethnic groups. The Minang people are Muslims and are the largest matrilineal society in the world [38]. In this matrilineal society, where women inherit the land and assets, they also play an important role in transmitting knowledge within the clan. In the Minangkabau food system, women play a crucial role in agricultural production and in the processing and preserving of food [39]. The Minangkabau have a rich knowledge and a natural philosophy related to agriculture and resource management, with concepts such as customary forests, protected waters, traditional agroforestry, planting trees after marriage and mutual cooperation [35,40]. 

Mandailing people had initially been a Batak sub-ethnic grow and were Christians until the 19th century when they converted to Islam and started to adopt elements of Minangkabau culture. In contrast to the Minang culture, they adhere to the patrilineal heritage system, and maintain their Mandailing language. The Mandailing community is often described as a hardworking agricultural society with indigenous traditions and community governance [37]. Their way of life is also very much tied to the land and particularly the paddy fields. Both communities are clan-based, where clans as social units play an essential role in socio-cultural issues and in the management of natural resources.

### 2.3. Study Ethics, Approach and Sampling

This study is a part of the broader Food, Agrobiodiversity and Diet (FAD) project which aimed to improve the food and nutrition security of the Minangkabau and Mandailing communities in the Pasaman regency by promoting the use of agrobiodiversity and traditional foods. The project was approved by the Indonesian Ministry of Research, Technology, and Higher Education (RISTEK). The methodology was further reviewed by the ethical committee of the University of Indonesia (UI) in Jakarta, and ethical clearance was obtained (No. protocol 18-03-0291). The research followed the Code of Ethics of the International Society of Ethnobiology and all informants were familiarized with the research objectives, methods and expected results. The free prior informed consent was obtained in a written form from all the individual respondents or their spouses. The data were interpreted anonymously. The project was aligned with the goals and policy of the Indonesian National Medium Term Development Plan (RPJMN) 2015–2019, in particular with a key strategy (c) to improve the quality and nutritional value of the Indonesian diet.

Having improved nutrition as an ultimate goal, our sampling targeted women at reproductive age (15–49 years old), as women represent a group vulnerable to malnutrition [41]. A stratified random sampling of cocoa farmers involved in the SCPP (Sustainable Cocoa Production Programme implemented in the study area by Swisscontact Indonesia) program was applied. We interviewed 200 women individually (100 women from each ethnic group). In addition, in-depth qualitative data were obtained through four focus group discussions (FGD) with 68 knowledgeable women participants. The sampling of FGD respondents was done purposively to select knowledgeable and active participants. Key farmers, husbands and children were allowed to join and complement the discussions whenever suitable and whenever accepted by the women participants. The Mandailing respondents were selected from the Padang Gelugur sub-district (Sontang and Bahagia villages) and Minangkabau respondents from the Simpang Alahan Mati sub-district (Simpang and Alahan Mati villages), as shown in Figure 1. The selection of these locations followed a recommendation of the local staff from SCPP, and it was based on the feasibility of the fieldwork, preserved landscape, and a need to improve the people’s nutritional status.

### 2.4. Individual Interviews and Plant Identification

Individual semi-structured interviews using questionnaires were conducted by trained data enumerators supervised by the principal investigator. The interviews started with capturing socio-economic characteristics, including questions for Progress out of Poverty Index for Indonesia [42]. These were followed by ethnobiological and anthropological methods including freelisting [43]. A Likert scale was used to record perceptions [44] and attitude statements [45]. The attitude statements were designed a priori with the local partners to fit the study context and objectives. In addition to interviews, the food system practices were documented via participant observation and by informal open-ended discussions. For ethnobiological plant inventory, often a husband contributed to the discussion of plant identity or took part in “Walks in the woods” to seek specimens [43]. Whenever possible, plant specimens were photo-documented and collected for later identification. Although the communities perceived mushrooms as wild vegetables, we excluded mushrooms in the study due to their limited availability during the fieldwork. Plant species were pre-identified in the field and determined taxonomically by botanists from the Faculty of Biology at Andalas University in Padang. The herbarium specimens were deposited in the herbarium of Andalas University (ANDA).

### 2.5. Focus Group Discussions

**Figure 2:**
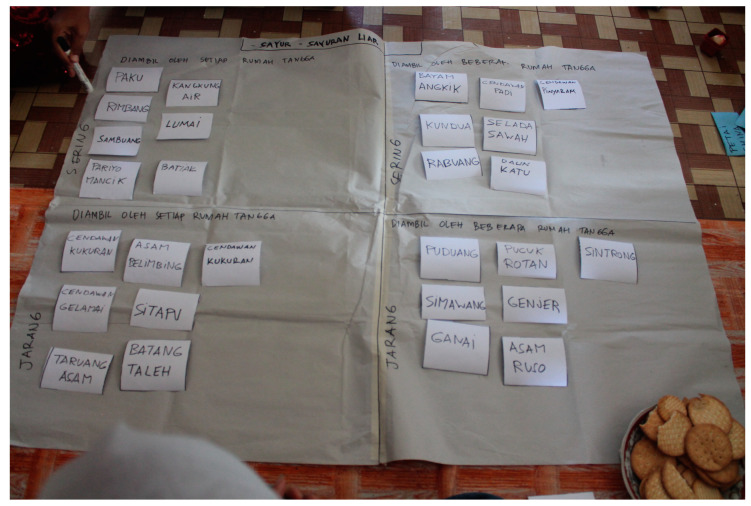
Participatory group assessment of changes in diversity and use of wild food plants through the 4-cell analysis method (Simpang village, June 2018).

Qualitative in-depth data on trends and changes in the plant use patterns were obtained through four focus group discussions (1 FGD per 1 village). In total, 68 women took part, sometimes accompanied by husbands or heads of farmer groups. A trained facilitator led the discussions following an open-ended questionnaire, while assistants took notes. Besides general questions and answers, we applied two main participatory exercises: seasonal crop calendars [46] and 4-cell analysis (Figure 2) [47]. The latter was the principal method of collecting data on changes in the use of WFPs along with motivations and barriers. Firstly, we prepared individual cards for each WFP, and women assessed the concurrent use of WFPs by sorting cards into four cells representing the different extents of plant use. Then we discussed contemporary barriers and motivations. Secondly, we asked women to re-organize the cards to show how the situation was in the past (around 20 years ago). After reshuffling, we asked the reasons for the change in use. With the women’s permission, the discussions were recorded by an audio recorder. 

### 2.6. Data Management and Analysis

After data cleaning, the individual and quantitative data were analyzed initially by functions and pivot tables in Microsoft Excel, followed by the descriptive and inference statistics performed in the IBM SPSS program version 22 (IBM Corp., Armonk, NY, USA). The comparison of means between the ethnic groups was made by the Mann–Whitney U test. The relationships between knowledge of WFPs and socio-ecological characteristics were assessed by multiple linear regression to identify the predictors of traditional knowledge on WFPs. The relationship of plant parts used with the extent of their use was visualized by an Alluvial diagram using RAWGraphs [48], while the importance of land-use systems as sources of WFPs was analyzed by Chord diagram in the R programming language (EthnobotanyR package [49]).

The qualitative data, such as the reasons for changes in the use of WFPs, were coded and categorized into emerging themes through inductive thematic analysis [50]. We opted for a posteriori inductive approach as it can better represent local views [51] and as the current food system framework does not align well with the context of consumers who are simultaneously also food producers or collectors. However, after categorizing the reasons into emerged themes, we followed the ecological framework on what people eat, developed by [52] to determine whether the reasons are related to personal factors, social environment, physical environment or macro-level. The changes in the use of WFPs were then discussed in the context of the systemic drivers [22]. The coding was conducted using the software ATLAS.ti version 7.5.18 (Scientific Software Development GmbH, Berlin, Germany).

WFPs were categorized into the food groups of dietary diversity [41]. The reason for following this grouping was that the overall project aimed to improve dietary diversity, and therefore it followed the nutritionally validated food groups. Nevertheless, the locally perceived categories were captured too.

## 3. Results

### 3.1. Contextualizing WFPs in the Minangkabau and Mandailing Food Systems

**Figure 3:**
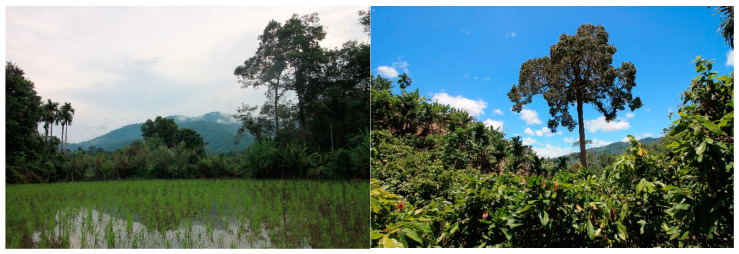
Traditional landscape in the study area (rice field in Simpang village on the left; cocoa agroforestry in Sontang village on the right, July 2017).

The traditional food system of the studied communities is strongly linked with rice production and with agroforestry gardens (Figure 3). Almost every household had these two principal land-use systems, which are used for their own food production as well as for income generation, with a highly varied ratio of subsistence to market orientation between the households. Food crops are also grown in home gardens (kitchen gardens) and occasionally in field plots and other lands not used for rice production. Crop diversity is generally high, and around half of the households raised farm animals, mostly chickens, and more rarely duck, fish or goat. Natural habitats such as forests, rivers, and streams are used to a smaller extent to acquire wild foods, mostly WFPs and various types of fish. The diet is dominated by a high intake of rice, accompanied by a small amount of vegetables and meat, mostly fresh or dried fish. Fruits are consumed irregularly and with high variation due to seasonality. The traditional foods contain lots of spices (mostly chili, onion and garlic) and many include coconut milk. WFPs are consumed to a small extent and rather spontaneously (based on our unpublished dietary assessment). In terms of food preparation, wild vegetables are consumed cooked, either stir-fried or boiled, whereas wild fruits are primarily consumed raw. WFPs are collected from both natural and managed lands, as well as purchased in traditional markets, where more and more households are purchasing foods. Considering the transition of food environments [53], the area can be characterized as an agrarian society with trade, as the main food environment is composed of wild and cultivated food environments and with regular informal markets composed mainly of wet markets and kiosks. Although the communities still prefer and consume traditional foods, the availability and consumption of fried snacks and ultra-processed foods is increasing.

### 3.2. Natural Food Environments as the Main Source of Wild Food Plants

**Figure 4:**
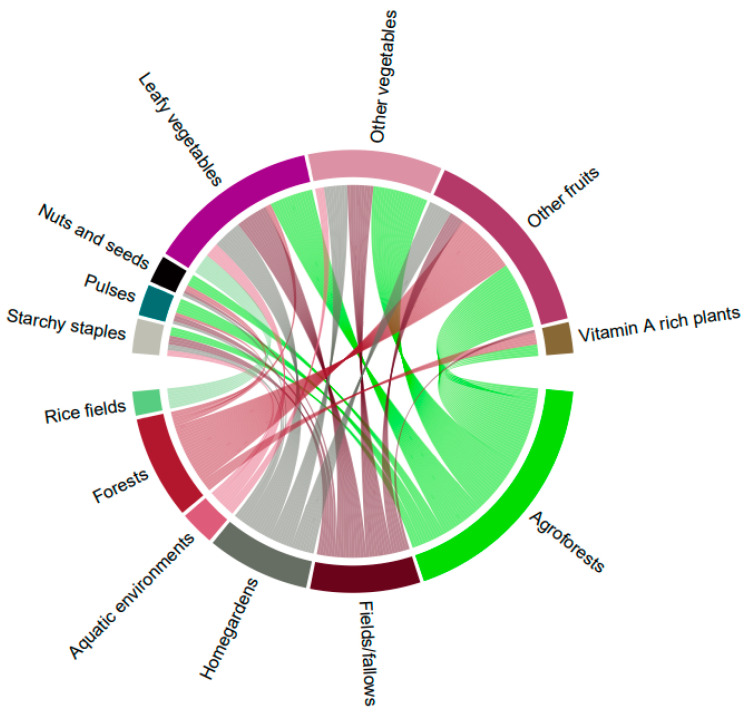
Land uses as sources of wild food plants in particular food groups (the thicker the stream, the more types of wild food plants are found in that land use).

Cocoa agroforests were the major sources of WFPs, where 74 WFPs were found. This is noteworthy as agroforests are managed lands showing that some amount of human disturbance can result in a greater diversity of useful plants than in purely wild habitats (see also [54,55]). After agroforests, the lands which were richest in WFPs were forests (40 species), fields (33 species) and home gardens (30 species). In contrast, aquatic environments (9 species) and rice fields (7 species) were less diverse, with only a few wild vegetables found. Figure 4 visualizes the biodiversity of WFPs in particular food groups across all the land-uses. We can see that agroforests are the most diverse and that WFPs from agroforests contribute to the following food groups: other fruits, other vegetables, and leafy vegetables; and to a lesser extent nuts and seeds, pulses and starchy staples. In view of food environment typology [53], local agroforests and other land-uses are not single but complex food environments, as they are a source of both wild and cultivated foods. Overall, the existence of WFPs is intertwined with local knowledge, traditional agriculture and landscape management.

### 3.3. Diversity of Wild Food Plants and Comparison of Knowledge between the Ethnic Groups

**Figure 5:**
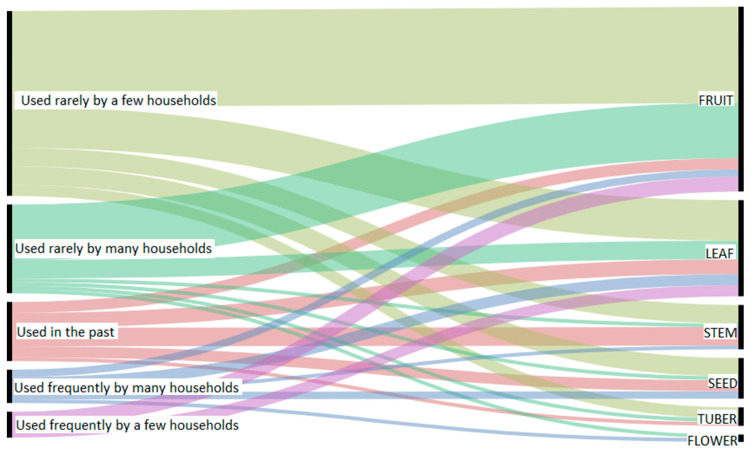
Plant parts used according to their extent of use (the thicker the stream, the more types of wild food plants).

The communities in Pasaman steward traditional knowledge on 106 WFPs, corresponding to 85 species, 65 genera and 37 botanical families (Table S1). The best-represented botanical families were Leguminosae (10 WFPs), Moraceae (7 WFPs) and Solanaceae, Araceae and Arecaceae (all by 6 WFPs). Concerning plant parts, the most prevalently used were fruits (48%, including unripe fruits used as vegetables), leaves (25%, including young shoots or tender leaf stems), seeds (10%), stems/shoots (11%, including palm hearts of 2 palm species), tubers (5%) and lastly flowers (2%). Figure 5 shows the plant parts used according to their extent of use. It can be seen that the most of WFPs are used for their fruits and leaves, which likely do not threated the survival of the plants. Use of underground organs would be more harmful [56], but in the study area, tubers of a few common species are used minimally or in the past. Figure 5 also demonstrates that the majority of households use WFPs rarely, some are not used anymore, and only a few of preferred WFPs are used frequently. 

The distribution of WFPs across the food groups demonstrated that the most diverse were other fruits (30 WFPs), followed by other vegetables (29 WFPs), leafy vegetables (27 WFPs), pulses (6 WFPs), nuts and seeds (5 WFPs), vitamin A-rich plants (5 WFPs) and lastly starchy staples with 4 WFPs. Comparison of traditional WFP knowledge between ethnic groups showed that Minangkabau women were familiar with 93 WFPs compared to 83 WFPs known by Mandailing women (Table 1). On average, Minang and Mandailing women listed 14.0 ± 6.9 and 10.2 ± 5.3 WFP species respectively. The difference in knowledge is statistically significant (*Z* = −4.145; *p* = 0.000). Minangkabau were found to know 23 unique food plants which do not occur in the Mandailing area, whereas the Mandailing community had only 13 unique food plants. Overall, two-thirds of WFP diversity (67% = 70 WFPs) overlapped and were common to both ethnic groups. We ran multiple linear regressions to determine the predictors of traditional WFP knowledge, but none of our social or ecological variables significantly predicted the knowledge of WFPs (*p* > 0.05). In the final model, all the variables together gave a weak correlation of *r* = 0.260, and they predicted the knowledge only by 7% (*R*^2^ = 0.07). However, as mentioned above, Minang women knew a significantly higher number of WFPs, which is likely related to the greater remoteness of Minang villages from the main road. In addition, the Minang are the dominant and are the ancestral group of West Sumatra, whereas the Mandailing arrived from North Sumatra more recently.

### 3.4. Perceptions and Attitudes Towards Wild Food Plants

**Figure 6:**
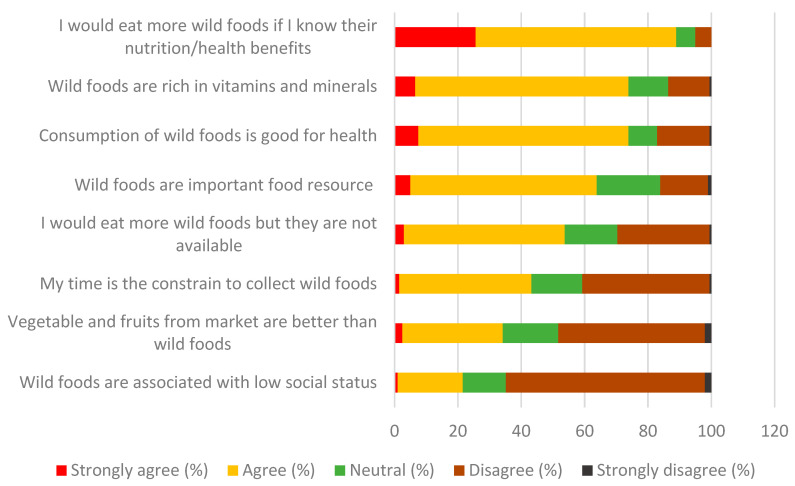
Attitudes of women towards consuming wild food plants.

Perceptions and attitudes are principal drivers of human behavior. We assessed attitudes towards wild and cultivated food plants using “barrier analysis statements” [45], adjusted to the study context and aims. A level of the agreement is given in Figure 6. The strongest agreement came with the statement “I would eat more wild foods if I knew their nutrition and health benefits”. This is followed by strong agreement with the statement “wild foods are rich in vitamins and minerals” and “Consumption of wild foods is good for health”. The strongest disagreement was found for the statement “Wild food plants are associated with lower social status”. From these attitudes, we can deduce that the majority of women perceived WFPs positively. They also assumed that WFPs are nutritious and healthy, but during group discussions, they mentioned a lack of information about their health benefits. Eliminating this knowledge gap would likely improve perception and consumption.

Using a similar method, but with a simplified 3-option scale, we compared the perception of WFPs versus commercial food plants from markets. The answers of 95% of women clearly showed that WFPs have a lower market value. On the other hand, the majority of women perceived WFPs to be tastier than the purchased plants (tastier = 63%, same = 24%, less tasty = 13%). Lastly, when asked about the image/prestige of wild and marketed plants, 47% of women considered them equal, 33% considered WFPs to be more prestigious and 20% considered them less prestigious. Overall, we can conclude that WFPs are perceived positively, but have a low economic value.

**Figure 7:**
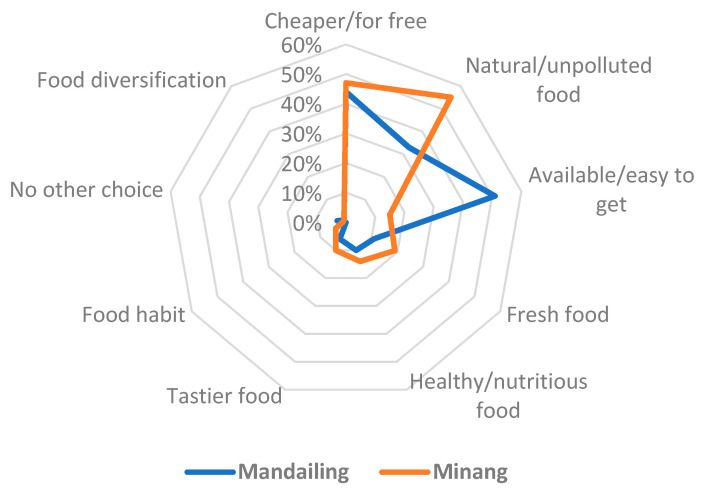
Motivations for consuming wild food plants (% of women).

To draw a full picture, we further let women list specific reasons for continuing the consumption of WFPs. What we found is that the most prevalent motivations were that WFPs are obtained for free or at a low cost (45%); that they are natural and unpolluted by agricultural chemicals (44%); and that some are still available and easy to obtain (32%) (Figure 7). While the economic factor (available for free) was of parallel importance for both ethnic groups, the importance of the availability was more prevalent among Mandailing women, while the aspect of being an unpolluted natural food was listed more by Minang women.

### 3.5. Trends in the Use of Wild Food Plants and Drivers of Change

In general, the results showed that the collection and consumption of WFPs has declined over the last generation. The reasons for the changes, as well as the barriers and motivations for contemporary use of WFPs, were categorized into the following six themes: (i) availability; (ii) livelihood and lifestyle; (iii) food, consumption, health; (iv) income, marketing, economy; (v) multifunctionality/processing; and (vi) knowledge and skills. Thematically categorized motivations and barriers to the current use of wild vegetables, along with reasons for a greater use of wild vegetables in the past, are given in Table 2.

The motivations and barriers to the contemporary use of wild fruits, along with reasons for a greater use of them in the past, are given in Table 3.

We grouped all the reasons related to both wild vegetables and fruits according to the emerged themes (called factors onwards). Each factor contains a paragraph on changes in the use of WFPs compared to the past, followed by information on contemporary barriers and motivations. The last paragraph discusses the changes on the ecological framework on what people eat [52] and attempts to identify the broader systemic drivers of changes. The findings are enriched by quotations from the respondents.

#### 3.5.1. Factors of Availability

Changes in the availability of WFPs were the most prevalent explanations for their decreased use. The most common reason was that WFPs were more abundant and easier to get in the past. Women further disclosed that in earlier times, the area was more forested and that people did not spray agricultural chemicals, which are now eradicating many WFPs and wild vegetables in particular. In addition, both gardens and landscapes were more spacious and more wild vegetables and fruits occurred there naturally. 

Currently, some WFPs are underutilized because they are not very available. Moreover, they are not so common in the markets, while other food options can be purchased or grown. Some wild fruits are now very rare or even extinct, and their presence is further undermined by spraying agricultural chemicals and removing shade trees. Lower availability of land has also become an issue.

However, some WFP species are still widely utilized, and availability plays a crucial role for the persistence in their use. Women mentioned specific motivations related to availability, such as that WFPs can be collected easily and on your own. Or that some WFPs are still plentiful in nearby lands, while others are only available further in the forest. Some women also mentioned that WFPs could be shared with other people. A few women explained that they are used to eating them when there are no other vegetables or fruits, especially in the lean season. 

The general decrease in the availability of WFPs can be attributed to the changes in the physical environment, which according to the responses, is caused mainly by the overuse of external inputs and changes in land management.

Minangkabau woman in Simpang village: “In the past, there were more forests, and people were collecting wild fruits and vegetables more. Now people use chemicals in the fields and wild food plants are gone”.

Mandailing woman in Sontang village: “Older people are more used to the taste of wild food plants from the past, well we like them too, the main issue is that they became rare and far”.

#### 3.5.2. Livelihood and Lifestyle Factors

Changes in livelihoods and lifestyles were also found to be common reasons for abandoning the use of WFPs. In the past, people were gathering plants more collectively and they were going to forests more frequently for non-timber forest products. Besides, more people were gardening and there were also more enthusiasts using WFPs. 

Currently, there is a reduced interest in some WFPs. People are now busier and there is not as much time as in the past. In addition, tastes started to change, especially with the younger generations and their less natural way of life.

Despite the generally negative impact of lifestyle changes on the use of WFPs, some people are still enthusiastic about WFPs and eat them quite regularly.

The lifestyle changes and convenience issues affecting the use of WFPs are happening at the individual (personal), social, and macro-level on the ecological framework on what people eat. They are likely driven by modern trends and changing socio-economic needs.

Mandailing woman in Sontang village: “Before people used to eat more wild food plants as there were less cultivated crops. Now more fruits and vegetables are being cultivated, traded and preferred in general.”

#### 3.5.3. Factors Related to Food, Consumption and Health

Women explained that the taste of WFPs were perceived to be better in the past. People also valued natural food and the health benefits of WFPs more. Some women mentioned that WFPs were common foods needed every day. 

Currently, many WFPs are not consumed much as their taste is less preferred and they have become foods that people eat occasionally. Sometimes, older people might like them more, but in general, people are consuming more cultivated plants and purchased foods.

However, there are large differences in the extent of using different species, and some WFPs are still eaten regularly as they are considered tasty, natural and healthy foods. Regarding wild fruits, it appears that they are more popular among children. Wild vegetables are perceived by women to be healthy and rich in nutrients.

The changes in use related to food, consumption and health belong to the personal factors and physical food environment in the ecological framework. Based on the responses, the changes in this theme appear to be driven mainly by changes in the markets and agriculture production.

Minangkabau woman in Alahan Mati village: “I continue eating wild food plants because they are rich in vitamins, tasty, and they do not contain pesticides”.

#### 3.5.4. Economic Factors

Many people stated that WFPs were cheap or free, which was even more important in the past. In the past, more people were also engaged in selling WFPs. Economic factors appear not to have changed the use of WFPs dramatically, and some women continue to sell or buy them, however, the number of people engaged in this is lower. Nowadays, cultivated plants and food products are being sold and bought more.

Women still value the fact that WFPs are free (this was the most frequent motivation listed by 45% women individually). Some women noted that several of these plants have an economic value and are still a source of income.

The discussed economic factors such as expenditures and income are related to the personal factors as well as to physical environment (markets). The current trend of selling and buying more cultivated plants or processed foods is also likely driven by changes in the markets, agricultural production, and a better livelihood opportunity.

Mandailing woman in Sontang village: “In the past, people did not need to buy fruits and vegetables on the market, but now it is easier to buy them rather than go to the forest”.

Minang woman in Simpang village: “Wild edibles are good because they are available and fresh natural food which is for free”.

#### 3.5.5. Factors Related to Processing/Multifunctionality

In the past, women were more used to traditional processing and the cooking of wild foods. Nowadays, some women still appreciate that WFPs can be processed and used according to taste and occasion. But generally, collecting and processing WFPs is considered more demanding, less convenient, and is done less frequently. Buying and cooking food ingredients purchased at the local market is considered to be more convenient and it is becoming more common. 

Interestingly, women pointed out that some species are used more because of their multiple benefits and also their medicinal value in some cases.

The changes in this theme are mostly related to convenience and skills which are belonging to the personal factors on the ecological framework. The reduced processing and cooking of WFPs is driven largely by changing lifestyles and markets.

Minangkabau woman in the Alahan Mati village: “The process of preparing and cooking wild vegetables takes a long time”.

#### 3.5.6. Factors Related to Knowledge and Skills

A generation ago, people had a richer knowledge of WFPs. Currently, while the common WFPs are known to everyone, some women are not familiar with the taste of some less common WFPs and others do not even know that certain WFPs are edible. 

A further barrier is the lack of knowledge on how to cook these traditional foods. Another reason mentioned by farmers was missing knowledge of how to cultivate wild plants. From these points we can see first that there is a loss of traditional knowledge on diversity and uses of some WFPs; second, that there is lack of knowledge on improved management, the domestication of these wild resources and little innovation of cooking or processing methods.

The issues with limited knowledge and skills can be considered personal factors. The weaker traditional knowledge seems to be caused by lack of its transmission driven by changes in lifestyles and food environment, whereas the lack of modern knowledge can be associated with gaps in the education system and a lack of relevant policies and innovations in science and technology.

Group of Minangkabau women: “We don’t know how to cultivate or manage some less common wild species, and only a few women know how to cook them”.

## 4. Discussion

### 4.1. Comparison of Wild Food Plants Diversity with Other Regions

The total number of 106 WFPs (85 species) documented represents a relatively high diversity. A recent study in a Mandailing community in North Sumatra showed that 106 food plant species are being used, including wild and cultivated ones [57]. Further in North Sumatra, Batak Toba people from Peadungdung village were found to use 44 species [58]. Towards the east of Indonesia, only 22 WFPs species were found to be used in Sasak cuisine on Lombok island [59], while in Bali, 86 species are used [60]. Ninety WFPs, a similar number as in our study, was found by Ogle and colleagues in the Mekong Delta in Vietnam [61] and also by Chauhan et al. [54] in the drier environment of Indian Gujarat. A diversity of 90–100 species of wild foods has been identified as an average for Asian and African agricultural and forager communities [20]. However, there are exceptions, such as in Meghalaya state of North-East India, where Sawian et al. [62] found 249 species in the markets of the Khasi tribe. In Thailand, Cruz-Garcia and Price [55] found from 87 to 252 WFP species. Kang et al. [63] found 185 WFP species from the Chinese Han. The overall diversity of WFPs in the study area is thus comparable to other regions, besides parts of India and tropical Thailand and China, where local communities tend to use greater diversity. The present study documented some lesser-known local food plants such as *Elateriospermum tapos* Blume, *Plukenetia corniculata* Sm., *Hornstedtia conica* Ridl., *Hornstedtia elongata* (Teijsm. and Binn.) K. Schum. or *Salacca sumatrana* Becc.

### 4.2. Local Perceptions and Attitudes on Wild Food Plants

The studied communities do not strictly divide between wild or cultivated plants, and in daily life, they call food plants by their vernacular names without further distinguishing. Sometimes they distinguish between traditional and modern food plants, where they use the term “local” for indigenous food plants and “modern/from the market” for the exotic and commercialized plants. Most respondents did not consider WFPs as “food of the poor”, a notion that has often developed in some regions [64] or “famine food” [65]. Minang and Mandailing communities perceived WFPs generally positively, appreciating the fact that they are a freely available, tasty, healthy and unpolluted food (people are concerned by heavy use of agrichemicals applied in commercial agriculture, and this to some extent, enhances the perception of local food plants). Other studies also noted positive attitudes towards WFPs. In both rural and urban Japan, WFPs were labelled as being a tasty, healthy and safe food [66]. WFPs were also perceived positively and as healthy elsewhere [67,68,69]. However, similar to other areas [54,70,71], the “change of taste” has started to occur among younger generations who interact less with nature and are more exposed to markets and more processed foods. As exotic species penetrate markets, in many places, traditional species become undervalued [20]. The increasing availability of processed and ultra-processed foods can result in a dietary transition with reduced dietary quality and rising rates of diet-related health problems [7]. It is also possible that dietary diversity can increase with markets, but this depends on affordability and food choices [72].

### 4.3. What Are the Reasons for the Decreased Use of Wild Food Plants?

Most of the available studies from various regions have found that socio-cultural factors are the main drivers of the reduced consumption of WFPs [70,73,74,75]. Here we find that instead, reduced availability was the most common factor limiting the consumption of WFPs in West Sumatra. This is similar to findings by Chauhan et al. [54], in Indian Gujarat and by Sõukand in rural Estonia [69].

Łuczaj et al. [64] showed that both social and ecological factors have reduced the use of wild plants in the European context (mainly the reduced contact with nature, and massive changes in agriculture and ecosystems). Apart from the biocultural refugia in mountainous areas or in the Mediterranean region, Europe has experienced a gradual disappearance of WFPs from diets [76]. The global trends and multiple factors are changing the use of wild plants across the world [20], including Africa (e.g., [13]) and America (e.g., [77]). Meanwhile, in Asia, the use of WFPs and particularly wild leafy vegetables appear more persistent [76]. The use of wild plants tends to persist among Indigenous communities [5]. In the studied sites in West Sumatra, although changes in lifestyle and perceptions have played a role in the reduced consumption of WFPs, they appear not to be the major drivers, as the traditional culture is still strong in the region. People still value and prefer their traditional foods, and diets are changing less dramatically in the region [78]. In this context, we found that changes in the availability of WFPs caused by agriculture intensification is the most important factor driving reductions in consumption. An increased use of chemical pesticides is known to eliminate not only pests and weeds, but overall field biodiversity too, including edible plants and animals [79]. Our respondents recalled a major decline in the diversity of edible weeds, fish and crustaceans in rice fields as they are now managed chemically by most of the farmers. Opportunities to earn more income along with supportive programs and markets drive this intensification of production.

Besides reduced availability, other reasons for not consuming WFPs were limited knowledge about their nutrition and health benefits, time involved to collect and prepare these foods and the lower economic value of these resources. Limited information on nutrient composition of wild foods is a well-known challenge [11,80] and more research, investment and mainstreaming are needed. Time constraints and convenience are related to lifestyle and livelihood changes [52], while the issue of the remoteness of wild foods was found in other countries too [69,70,81]. The low economic value of WFPs is common in other regions (e.g., [21]), but it does not appear as a main driver of change in the study area. 

FAO [22] identified the most widespread threats to WFP use as overexploitation, habitat alteration, pollution, land-use change and deforestation. Some of these issues might also be factors here, but were not perceived by respondents. 

### 4.4. What Motivates People to Continue Consumption of Wild Food Plants?

Understanding the motivations of human behavior can enable us to design more effective solutions to achieve the needed changes. More studies have looked at the reasons for the decrease in WFPs, as opposed to the actual motivations for their continued consumption. This is likely due to the global downward trend in the use of WFPs. Despite the overall decreasing trend, some studies in Himachal Pradesh [70], North-Eastern Thailand [82], Estonia [69] and the Catalan Pyrenees and Balearic Islands of Spain [75] found the taste of WFPs to be the primary motivation for their continued consumption. In more industrialized countries or regions, the motivations for the use of WFPs have moved towards recreation or seeking innovative food trends [74,83,84], whereas, in more traditional and indigenous territories, wild resources play a more critical dietary, economic and cultural role [5]. In the area studied, the primary motivations for the use of WFPs were that they were freely available and that they are considered unpolluted natural foods (see Figure 7 for all the motivations). Nevertheless, we found substantial differences in the characteristics and use of individual plant species, where some WFPs are used more than others because of their higher availability, better taste, larger size, easier management or collection and their multiple uses or economic value. Indeed, a whole range of factors determines whether the particular species is better utilized, underutilized or abandoned. Future interventions may need to consider these differences and prioritize locally preferred species with a higher potential for wider use. 

### 4.5. Need for an Integrated Approach for Sustainable Use of Wild Food Plants

Public health policy across many countries tends to operate within a model of food security and nutrition that discounts the biodiversity and traditional food practices of the communities [85]. Moreover, other policies and sectors have overlooked these existing resources, which means missed opportunities and eventually the implementation of more costly or less sustainable interventions. Numerous scientists and international conventions have recognized the importance or potential of WFPs for food and nutrition, e.g., Global Strategy for Plant Conservation of the Convention on Biological Diversity (CBD), the International Treaty on Plant Genetic Resources for Food and Agriculture, or the Second Global Plan of Action for Plant Genetic Resources for Food and Agriculture. Now more action should be taken at the national and local levels. 

What needs to be recognized is that WFPs have to be sustained in a participatory manner with local communities and their evolving intercultural knowledge. This inclusive approach of food biodiversity conservation through local knowledge and practices allows the continuous evolution and adaptation to socio-ecological change [86,87]. To guide countries and different stakeholders in developing plans and strategies related to WFPs, Borelli et al. [88] proposed an integrated approach for using and conserving WFPs. That approach calls for and guides the many stakeholders and actors to take action to ensure that WFPs are used sustainably and maintained for future generations.

The sustainability of use is an important aspect that needs to be considered where wild plants are being used and particularly when they are being promoted. Overharvesting or using whole plants or certain plant parts such as roots can have strong implications for the existence of these resources and should be avoided [56]. In the study area, rather than overharvesting, there is a general trend of decline in intensity of WFP use. The availability of WFPs is decreasing due to broader drivers such as land and agriculture intensification. 

**Figure 8:**
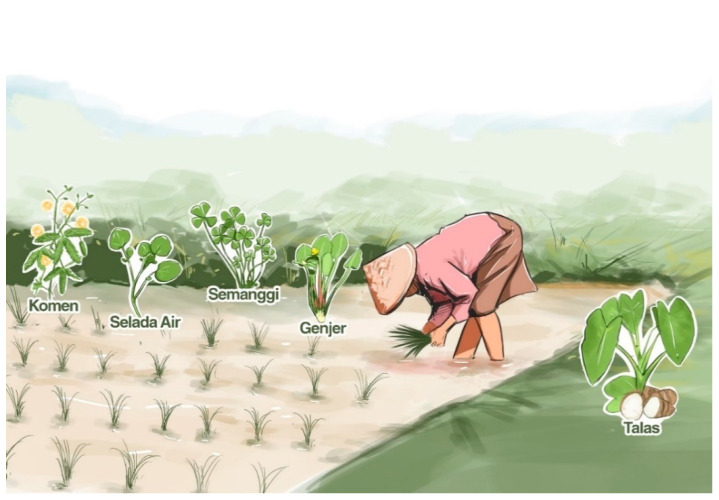
Illustration of wild vegetables naturally occurring in the organically managed rice field (adapted from the community guidebook by Pawera et al. [89]).

In the context of scaling sustainable use of WFPs in Indonesia, the country could build on the previous work of PROSEA (http://proseanet.org/prosea/), existing food, ethnobiological and biodiversity studies, and conduct food mapping or barrier analysis where knowledge gaps exist. This should include an innovative action bringing the knowledge to the broader public and supporting actions at the local level. Figure 8 shows an example of illustration developed by the FAD project to raise awareness on WFPs. The project also produced a policy brief and a community guidebook on food plants for nutrition and health [89] (which can be requested from the first author). Examples of follow-up actions could be to integrate traditional and modern knowledge of WFPs into programs of education, tourism, certification schemes, community health workers “posyandu”, women groups and extension services. The government and stakeholders could support and incentivize local actions related to agrobiodiversity demo plots, home gardens, school gardening, food festivals, culinary tourism, rural-urban supply chains, traditional product development, community forestry and agroecological production.

## 5. Conclusions

Identifying interventions to improve diet and nutrition in Indonesia is one of the key issues for contemporary research and development in the country. Mainstream research and development, however, have been overlooking the potential of agrobiodiversity and WFPs, even though they could contribute to diversifying diets and provide functional foods, particularly to marginalized and vulnerable communities. The locations in this study were found to be still relatively rich in WFPs due to the persistence of traditional land-use systems and strong local culture. However, consumption of WFPs has declined over the last generation, despite the overall positive perception of these foods. The reasons for this decline were foremost their decreased availability (mainly due to agriculture intensification at the farm level) and changes in perceptions and lifestyle. The main overall drivers of change appear to be socio-economic factors, agriculture intensification and changing markets. On the ecological framework on what people eat [52], most of the changes relate to the personal factors and the physical food environment. The main contemporary barriers to consuming WFPs were their low availability, time constraints and the limited knowledge of their nutritional benefits. In contrast, the key motivations for their continued use were that they are freely available, are natural foods free of chemicals, and that some species are still abundant. 

The findings inform us what barriers and motivations can be acted upon to counteract underutilization and loss of this food biodiversity. This study found large differences in use and valuation of individual species, and a whole range of factors affecting whether the species is utilized, underutilized or abandoned. The fact that the local communities perceive WFPs positively offers an important opportunity for their successful promotion. This should be supported by actions for increasing their availability and raising awareness to fill the knowledge gap about their benefits. Both traditional and modern knowledge of these foods could be integrated into agriculture, food, nutrition, health, tourism, social and education programs in the area. There is a need for communities, government and NGOs to come together to undertake creative action to optimize the use of WFPs in an inclusive and sustainable way. This integrated approach of “conservation through use” could improve nutrition and health while conserving biodiversity and traditional knowledge.

## Figures and Tables

**Table 1:** Comparison of wild food plant diversity between Minangkabau and Mandailing ethnic groups.

Food Group	Total No. of WFPs	No. of WFPs in Minangkabau	No. of WFPs in Mandailing	No. of WFPs Unique to Minangkabau	No. of WFPs Unique to Mandailing	No. of WFPs Overlapping in Both
Starchy staples	4	4	3	1	0	3
Leafy vegetables	27	22	23	4	7	16
Other vegetables	29	25	22	7	4	18
Pulses	6	5	5	1	1	4
Nuts and seeds	5	4	4	1	1	3
Vitamin A rich plants	5	5	4	1	0	4
Other fruits	30	30	22	8	0	22
Total	106	93	83	23	13	70

WFPs = wild food plants.

**Table 2:** Barriers, motivations and reasons for the changes in the use of wild vegetables.

Theme	Reasons for Using Wild Vegetables More in the Past	Reasons for Underutilizing Selected Wild Vegetables Currently (Barriers)	Reasons for a Greater Use of Selected Wild Vegetables Currently (Motivations)
Availability	Easy to get (Mi, Ma) Still plenty of them (Mi) There were no other vegetables (Mi, Ma) Abundant forests (Mi, Ma) Spacious gardens (Mi) Collect their own (Ma)	Competitiveness (Mi, Ma) Not available in the market (Mi) Hard to get (Mi, Ma) Limited land (Mi) Not much available (Ma)	Can be obtained in the forest (Mi) Can be shared (Mi) There are no other vegetables (Mi) Land area available (Mi) Easy to get (Ma) At close range (Ma)
Livelihood and lifestyle	Community collection (Mi) People were gardening more (Mi) People were often going to the forest (Ma) Many enthusiasts (Ma)	Reduced interest (Mi) Not everyone likes it (Ma)	Many enthusiasts (Mi, Ma)
Food, consumption, health	People liked them (Mi) Food was needed every day (Ma) Healthy (Ma)	Taste disliked (Mi, Ma) Not consumed much (Ma)	People like them (Mi, Ma) These are required and eaten regularly (Mi, Ma) Rich in nutrients (Ma)
Income, marketing, economy	They are free (Mi, Ma)		No need to buy (Mi, Ma) Good economic value (Mi) Source of income (Mi)
Multifunctionality/processing	Easy to grow (Mi) Easy processing (Mi) Traditional processing (Ma)	Need good care (Mi) Processing is not easy (Mi)	Good benefits (Mi) Multiple benefits (Ma)
Knowledge and skills		Don’t know the taste (Mi) Don’t know that they can be consumed (Mi) Don’t know how to cook them (Ma)	

Mi = Minangkabau; Ma = Mandailing.

**Table 3:** Barriers, motivations and reasons for the changes in the use of wild fruits.

Theme	Reasons for Using Wild Fruits More in the Past	Reasons for Underutilizing Selected Wild Fruits Currently (Barriers)	Reasons for Greater Use of Selected Wild Fruits Currently (Motivations)
Availability	There were no other fruits (Mi, Ma) Seasonal (Mi, Ma) Many were available (Mi, Ma) Easy to collect or grow (Mi, Ma) People did not spray chemicals (Ma) Land was available (Ma)	Rare or extinct (Mi, Ma) Grow in the forest (Mi) They are only seasonal (Mi, Ma) Depends on the land (Mi) Hard to get (Mi, Ma) Decreasing from spraying agrichemicals (Ma) Not in the market (Ma) Difficult to cultivate (Ma)	There are no other fruits (Mi, Ma) Can be collected on your own (Mi) Easy to collect (Ma) Still plentiful (Ma)
Livelihood and lifestyle	People often went to the forest (Mi)	Not a big interest (Mi) People are busy and lack of time (Ma)	Many enthusiasts (Mi)
Food, consumption, health	People liked the taste (Mi, Ma) Natural and healthy (Ma)	Not so tasty (Mi) Taste preferences have changed (Ma)	They are tasty (Mi) Eaten every day (Mi) They are needed (Mi) Many people like it (Ma) Kids like them (Ma)
Income, marketing, economy	Can be sold (Mi) Cheap to purchase (Mi, Ma) No need to buy (Ma)		Can be sold (Mi) No need to buy (Ma)
Multifunctionality/processing		Used also as a medicine (Mi)	Can be cooked according to taste (Mi)
Knowledge and skills		Don’t know how to cultivate them (Mi) We don’t know them (Mi)	

Mi = Minangkabau; Ma = Mandailing.

## Supplementary Materials

The following are available online at https://www.mdpi.com/2304-8158/9/9/1240/s1, Table S1: Wild food plants used by Minangkabau and Mandailing women in Pasaman regency, West Sumatra, Indonesia.
