# Supplementary material for: Wild Food Plants and Trends in Their Use: From Knowledge and Perceptions to Drivers of Change in West Sumatra, Indonesia
Source: Foods. 2020 Sep 4;9(9):1240. doi: 10.3390/foods9091240 (PMC7555794; doi:10.3390/foods9091240)
Supplement: Supplementary file 1 [file foods-09-01240-s001.pdf]

Table S1 Wild food plants used by Minangkabau and Mandailing women in Pasaman regency, West Sumatra, Indonesia

Plant species and voucher number	Plant family	Local names	Local food category	Part used and extent of use	Cited by % of respondents	Habitat
**Food group: Starchy staples**						
Manihot esculenta C rantz	Euphorbiaceae	Ubi singkong, Ubi kayu (Mi, Ma)	Staple food/snack	Tuber ++	74 (30 Ma; 44 Mi)	Ag, Ho, Fi
Colocasia esculenta (L.) Schott (LP16)	Araceae	Talas (Mi); Suhat (Ma)	Staple food/snack	Tuber +	53 (16 Ma; 37 Mi)	Ae, Af, Fi
Ipomoea batatas (L.) Poir.	Convolvulaceae	Ubi jalar (Mi, Ma)	Staple food/snack	Tuber +	25 (30 Ma; 44 Mi)	Fi, Hg
Xanthosoma sagittifolium (L.) Schott (LP56)	Araceae	Talas hitam (Mi)	Staple food/snack	Tuber -	1 (0 Ma; 1 Mi)	Af
**Food group: Pulses**						
Archidendron pauciflorum (Benth.) I.C.Nielsen	Leguminosae	Jariang (Mi); Joring (Ma); Jengkol (Mi, Ma)	Vegetable	Seed ++++	14 (4 Ma; 10 Mi)	Af
Parkia speciosa Hassk.	Leguminosae	Petai (Mi, Ma)	Vegetable	Seed ++++	7 (4 Ma; 3 Mi)	Af
Archidendron bubalinum (Jack) I.C.Nielsen	Leguminosae	Kabau, Sikabau (Mi); Kaladeh (Ma)	Vegetable	Seed ++	3 (0 Ma; 3 Mi)	Af
Parkia speciosa Hassk. (LP17)	Leguminosae	Potar, Parira, Petai hutan (Ma)	Vegetable	Seed +	1 (1 Ma; 0 Mi)	Af, Fo
Species not identified (LP41)	Leguminosae	Kacang tujuh lembar daun (Mi)	Vegetable/ bean	Seed +	0 (0 Ma; 1 Mi)	Af, Fi
Vigna unguiculata 'kacang tunjuk' (LP35)	Leguminosae	Kacang tunjuk (Mi, Ma)	Vegetable/ bean	Seed -	Only FGD (Mi, Ma)	Fi, Hg
**Food group: Nuts and Seeds**						
Artocarpus sp.	Moraceae	Nankga hutan (Mi); Nangka/Sibodak rimbo (Ma)	Vegetable	Fruit (unripe) +	13 (3 Ma; 10 Mi)	Fo
Pangium edule Reinw.	Achariaceae	Siwamang (Mi); Hapesong (Ma)	Fruit	Seed +	2 (0 Ma; 2 Mi)	Af
Gnetum gnemon L. (LP53)	Gnetaceae	Melinjo (Mi, Ma)	Fruit	Seed +	Only FGD (Mi, Ma)	Af, Fi
Elateriospermum tapos Blume (LP43)	Euphorbiaceae	Tapuih (Mi)	Fruit	Seed -	Only FGD (Mi)	Af, Fo
Artocarpus altilis (Parkinson ex F.A.Zorn) Fosberg	Moraceae	Hunur (Ma)	Vegetable	Seed +	0 (1 Ma; 0 Mi)	Af, Hg
**Food group: Leafy vegetables**						
Diplazium esculentu m (Retz.) Sw. (LP08)	Woodsiaceae	Pahu (Mi); Pakis (Ma)	Vegetable	Leaf ++++	163 (69 Ma; 94 Mi)	Ae, Fo

Table S1 Continued

Plant species and voucher number	Plant family	Local names	Local food category	Part used and extent of use	Cited by % of respondents	Habitat
Ipomoea aquatica Forssk.	Convolvulaceae	Kangkung air Kangkuang (Mi); Kengkong, Kangkung (Ma)	Vegetable	Leaf, stem ++++	122 (73 Ma; 49 Mi)	Ae, Rf
Manihot esculenta Crantz	Euphorbiaceae	Pucuk ubi, Daun singkong (Mi, Ma)	Vegetable	Leaf ++++	77 (36 Ma; 41 Mi)	Ag, Fi, Hg
Solanum americanum Mill. (LP19)	Solanaceae	Lumai (Mi); Ranti (Ma)	Vegetable	Leaf, stem ++	76 (44 Ma; 32 Mi)	Ag, Fi, Hg
Limnocharis flava (L.) Buchenau	Alismataceae	Genjer (Mi), Kalanyoe (Ma)	Vegetable	Leaf, stem ++	60 (32 Ma; 28 Mi)	Ae, Rf
Amaranthus hybridus L. (LP49)	Amaranthaceae	Bayam, Bayam liar (Mi); Siarum (Ma)	Vegetable	Leaf, stem +++	42 (30 Ma; 12 Mi)	Af, Fi, Hg
Carica papaya L.	Caricaceae	Daun papaya (Mi), Bulung botik (Ma)	Vegetable	Leaf +++	34 (18 Ma; 16 Mi)	Af, Fi, Hg
Claoxylon longifolium (Blume) Endl. ex Hassk. (LP28)	Euphorbiaceae	Sitopu, Sitapu (Mi, Ma); Daun manis (Ma)	Vegetable	Leaf ++	23 (6 Ma; 17 Mi)	Af
Sauropus androgynus (L.) Merr.	Phyllanthaceae	(Daun-) Katuk, Taruak manih (Mi); Nasi-nasi (Ma)	Vegetable	Leaf +++	20 (13 Ma; 7 Mi)	Af, Fi, Hg
Neptunia oleracea Lour. (LP15)	Leguminosae	Komen, Koman (Ma)	Vegetable	Leaf, stem ++	13 (13 Ma; 0 Mi)	Ae, Rf
Moringa oleifera Lam. (LP22)	Moringaceae	(Daun-) Kelor (Mi), Barrunge (Ma)	Vegetable	Leaf ++	10 (10 Ma; 0 Mi)	Hg, Fi
Rorippa indica (L.) Hiern (LP30)	Brassicaceae	Lobak kampung/ lokal (Mi, Ma)	Vegetable	Leaf +	6 (3 Ma; 3 Mi)	Af, Fi
Plukenetia corniculata Sm.	Euphorbiaceae	Pina-Pina (Ma)	Vegetable	Leaf +	6 (6 Ma; 0 Mi)	Af
Brassica spp.	Brassicaceae	Sawi, Sabi (Mi, Ma)	Vegetable	Leaf ++	6 (3 Ma; 3 Mi)	Af, Fi
Lactuca indica L. (LP23)	Compositae	Sijungkat (Ma)	Vegetable	Leaf +	4 (4 Ma; 0 Mi)	Af, Fi
Amaranthus sp. (LP31)	Amaranthaceae	Bayam angkik/ duri/putih (Mi, Ma)	Vegetable	Leaf, stem +	3 (3 Ma; 1 Mi)	Af, Fi, Hg
Syzygium malaccense; *Syzigium* sp.	Myrtaceae	Pucuk jambak (Mi, Ma)	Vegetable	Leaf, shoot +	3 (0 Ma; 3 Mi)	Af, Hg
Cucurbita moschata Duchesne	Cucurbitaceae	Pucuk labu (Mi); Bulung jelok (Ma)	Vegetable	Leaf, shoot +	3 (2 Ma; 1 Mi)	Af, Fi, Hg
Nasturtium officinale R.Br.	Brassicaceae	Sayur paret, Selada sawah (Mi)	Vegetable	Leaf -	3 (0 Ma; 3 Mi)	Rf
Hydrocotyle javanica Thunb. (LP48)	Araliaceae	Tubo aie/aek, Pegagan (Mi)	Vegetable	Leaf -	2 (0 Ma; 2 Mi)	Rf
Hibiscus sabdariffa L.	Malvaceae	Asam ruso (Mi)	Vegetable	Leaf +	1 (0 Ma; 1 Mi)	Hg
Gnetum gnemon L. (LP53)	Gnetaceae	Daun melinjo (Ma)	Vegetable	Leaf +	Only FGD (Ma)	Af, Fi

Table S1 Continued

Plant species and voucher number	Plant family	Local names	Local food category	Part used and extent of use	Cited by % of respondents	Habitat
Sechium edule (Jacq.) Sw.	Cucurbitaceae	Bulung jepang (Ma)	Vegetable	Leaf, shoot<br>+	1 (1 Ma; 0 Mi)	Af, Hg
Marsilea polycarpa Hook. & Grev. (LP13)	Marsileaceae	Simmange (Mi); Simmangah (Ma)	Vegetable	Leaf<br>-	1 (1 Ma; 0 Mi)	Rf
Alternanthera sessilis (L.) R.Br. ex DC. (LP34)	Amaranthaceae	Kagama (Mi)	Vegetable	Leaf<br>-	Only FGD (Mi)	Rf
Cyathea junghuhniana (Kunze) Copel. (LP07)	Cyatheaceae	Pahu (Mi); Pakis (Ma)	Vegetable	Leaf<br>+	0 (1 Ma; 0 Mi)	Ae, Af, Fo
Oxalis barrelieri L. (LP26)	Oxalidaceae	Sayur asam (Ma)	Vegetable	Leaf<br>-	Only FGD (Ma)	Af, Fi
**Food group: Other vegetables**						
Bambusa vulgaris Schrad. (LP12); *Gigantochloa atter* (Hassk.) Kurz (LP40)	Poaceae	Rabuang (Mi); Robung (Ma)	Vegetable	Shoot<br>++++	112 (41 Ma; 71 Mi)	Af, Fo
Solanum rudepannum Dunal	Solanaceae	Rimbang (Mi, Ma); Campur-campur (Ma)	Vegetable	Fruit<br>++++	87 (31 Ma; 56 Mi)	Af, Fi, Hg
Etlingera elatior (Jack) R.M.Sm. (LP02)	Zingiberaceae	Kimcuang, Sambuang (Mi); Kimcong (Ma)	Vegetable	Flower/fruit<br>++++	57 (6 Ma; 51 Mi)	Af, Fo
Calamus exilis Griff. (LP24)	Arecaceae	Pucuk rotan (Mi); Pangkat (Ma)	Vegetable	Shoot<br>++	22 (21 Ma; 1 Mi)	Fo
Momordica charantia L. (LP06)	Cucurbitaceae	Paria/Pariyo/Pare liar (Mi); Paria, Paria-paria liar (Ma)	Vegetable	Fruit<br>++	19 (5 Ma; 14 Mi)	Af, Fi
Solanum ferox L. (LP33)	Solanaceae	Torung asam (Mi, Ma)	Vegetable	Fruit<br>++	18 (1 Ma; 17 Mi)	Af
Artocarpus sp.	Moraceae	Nankga hutan (Mi); Nangka rimbo (Ma)	Vegetable	Fruit (unripe)<br>+	13 (3 Ma; 10 Mi)	Fo
Solanum lycopersicum LAM.	Solanaceae	Tomat liar (Mi, Ma)	Vegetable	Fruit<br>++	12 (11 Ma; 1 Mi)	Fi, Hg
Colocasia esculenta (L.) Schott (LP57)	Araceae	Batang talas (Mi, Ma); Suhat (Ma)	Vegetable	Stem<br>+	6 (1 Ma; 5 Mi)	Af, Ae, Fi
Sechium edule (Jacq.) Sw.	Cucurbitaceae	Labu jepang/siam (Mi); Bulung Jepang, Jepang (Ma)	Vegetable	Fruit<br>++	5 (3 Ma; 2 Mi)	Af, Hg
Momordica charantia subsp. *abbreviata* (Ser.) Greb. (LP37)	Cucurbitaceae	Pariyo, Pio mancik (Mi)	Vegetable	Fruit<br>+	4 (0 Ma; 4 Mi)	Af, Fi
Carica papaya L.	Caricaceae	Pepaya mudah (Mi, Ma); Botik mudah (Ma)	Vegetable	Fruit (unripe)<br>+	3 (1 Ma; 2 Mi)	Af, Fi, Hg

Table S1 Continued

Plant species and voucher number	Plant family	Local names	Local food category	Part used and extent of use	Cited by % of respondents	Habitat
Musa × paradisiaca L.; *Musa* sp.	Musaceae	Jantung pisang, Tukua pisang (Mi); Jattung pisang (Ma)	Vegetable	Flower<br>++	2 (0 Ma; 2 Mi)	Af, Hg
Arenga obtusifolia Mart. (LP25)	Arecaceae	Umbut puli (Ma)	Vegetable	Palm heart<br>+	2 (0 Ma; 2Mi)	Fo
Species not identified	Arecaceae	Umbut baih/langkok (Mi)	Vegetable	Palm heart<br>+	2 (0 Ma; 2 Mi)	Fo
Averrhoa bilimbi L.	Oxalidaceae	Asam belimbing (Mi, Ma); Belimbing besi (Mi)	Vegetable	Fruit<br>+	1 (0 Ma; 1 Mi)	Af, Hg
Colocasia spp.	Araceae	Batang/Taleh keladi (Mi); Keladi, Suhat (Ma)	Vegetable	Stem<br>+	1 (0 Ma; 1 Mi)	Ae, Af
Xanthosoma sagittifolium (L.) Schott (LP56)	Araceae	Batang talas hitam (Mi)	Vegetable	Stem<br>-	1 (0 Ma; 1 Mi)	Ae, Af
Pachyrhizus erosus (L.) Urb. (LP03)	Leguminosae	Benkuang (Mi, Ma)	Vegetable	Tuber<br>+	1 (1 Ma; 0 Mi)	Af
Psophocarpus tetragonolobus (L.) DC.	Leguminosae	Kacang belimbing (Mi); Kacang jorbing (Ma)	Vegetable	Fruit<br>+	1 (0 Ma; 1 Mi)	Af, Hg
Leucaena leucocephala (Lam.) de Wit	Leguminosae	Petai cina (Mi, Ma)	Vegetable	Fruit<br>+	1 (1 Ma; 0 Mi)	Af, Fi, Hg
Saccharum spontaneum L. (LP01)	Poaceae	Pira tobu, Tebu hitam (Ma)	Vegetable	Stem<br>+	1 (1 Ma; 0 Mi)	Af, Fi
Artocarpus sp.	Moraceae	Hunur (Ma)	Vegetable	Fruit<br>+	1 (1 Ma; 0 Mi)	Ae, Af
Colocasia gigantea (Blume) Hook.f. (LP39)	Araceae	Batang/Taleh Kemumu (Mi)	Vegetable	Stem<br>-	Only FGD (Mi)	Ae, Af, Fi
Carica papaya L.	Caricaceae	Bunga papaya (Mi, Ma); Bunga botiak (Ma)	Vegetable	Flower<br>+	Only FGD (Mi, Ma)	Af, Fi, Fo
Ananas comosus (L.) Merr.	Bromeliaceae	Naneh/Nenas mudo (Mi)	Vegetable	Fruit (unripe)<br>+	Only FGD (Mi)	Af, Fi, Hg
Musa acuminata Colla	Musaceae	Pisang hutan (Ma)	Vegetable	Stem<br>-	Only FGD (Ma)	Fo
Calamus manan Miq. (LP38)	Arecaceae	Tabu-tabu (Mi)	Vegetable	Shoot<br>-	Only FGD (Mi)	Fo
**Food group: Other fruits**						
Baccaurea racemosa (Reinw. ex Blume) Müll.Arg. (LP11)	Phyllanthaceae	Kapunduang (Mi, Ma)	Fruit	Fruit<br>+++	87 (37 Ma; 50 Mi)	Af, Fo
Durio zibethinus L., *Durio* sp. (LP59)	Malvaceae	Durian hutan/liar/batu/mangko (Mi; Ma)	Fruit	Fruit<br>+++	82 (38 Ma; 44 Mi)	Fo

Table S1 Continued

Plant species and voucher number	Plant family	Local names	Local food category	Part used and extent of use	Cited by % of respondents	Habitat
Psidium guajava L.	Myrtaceae	Jambu biji (Mi, Ma); Jambu paraweh (Mi); Jambu orsik (Ma)	Fruit	Fruit +++	40 (20 Ma; 20 Mi)	Af, Fo, Hg
Nephelium lappaceum L. (LP20)	Sapindaceae	Rambutan hutan/liar (Mi, Ma)	Fruit	Fruit ++	37 (22 Ma; 15 Mi)	Fo
Lansium parasiticum var. *Do mesticum*	Meliaceae	Langsek (Mi); Latcat, Langsat (Ma)	Fruit	Fruit ++	35 (11 Ma; 24 Mi)	Af, Fo, Hg
Clidemia hirta (L.) D. Don (LP27)	Melastomataceae	Karamunting (Mi); Haramunting (Ma)	Fruit	Fruit ++	29 (7 Ma; 22 Mi)	Af, Fi, Fo
Flacourtia rukam Zoll. & Moritzi (LP44)	Salicaceae	Ukam, Rukam (Mi)	Fruit	Fruit +	25 (3 Ma; 22 Mi)	Af, Fo
Hornstedtia conica Ridl. (LP51)	Zingiberaceae	Kudaro (Mi)	Fruit	Fruit +	24 (0 Ma; 24 Mi)	Af, Fo
Artocarpus heterophyllus Lam.	Moraceae	Nangka (Mi, Ma)	Fruit	Fruit +	22 (7 Ma; 15 Mi)	Fo
Species not identified		Bonai, Limpanai (Mi)	Fruit	Fruit +	21 (0 Ma; 21 Mi)	Af, Fo
Hornstedtia elongata (Teijsm. & Binn.) K.Schum.	Zingiberaceae	Puah, Puahtok (Mi)	Fruit	Fruit +	20 (0 Ma; 20 Mi)	Af, Fo
Salacca sumatrana Becc.	Arecaceae	Salak lokal/liar/hutan (Mi, Ma)	Fruit	Fruit ++	20 (6 Ma; 14 Mi)	Af, Fo
Syzygium aqueum (Burm.f.) Alston (LP04)	Myrtaceae	Jambu air, Jambu madu (Mi); Jambu aek/aie (Ma)	Fruit	Fruit ++	18 (10 Ma; 8 Mi)	Af, Fo, Hg
Melastoma malabathricum L. (LP18)	Melastomataceae	Sikaduduak (Mi); Sikaduduk (Ma)	Fruit	Fruit‘ ++	15 (3 Ma; 12 Mi)	Af, Fi, Fo
Baccaurea motleyana (Müll.Arg.) Müll.Arg. (LP47)	Phyllanthaceae	Rambai, Rumbai (Mi), Rambe (Ma)	Fruit	Fruit +	14 (9 Ma; 5 Mi)	Af, Fo
Rubus buergeri Miq. (LP61)	Rosaceae	Cimparingek, Lancinek, Cilinek (Mi)	Fruit	Fruit +	12 (0 Ma; 12 Mi)	Af, Fo
Calamus spp.	Arecaceae	Buah rotan (Mi); Sihim (Ma)	Fruit	Fruit +	11 (7 Ma; 4 Mi)	Fo
Lansium parasiticum var. *duku*	Meliaceae	Duku (Mi, Ma)	Fruit	Fruit +	11 (5 Ma; 6 Mi)	Af, Fo
Spondias dulcis Parkinson	Anacardiaceae	Kedongdong (Mi, Ma)	Fruit	Fruit +	11 (10 Ma; 1 Mi)	Af, Fo
Musa x *paradisiaca* L.	Musaceae	Pisang (Mi, Ma)	Fruit	Fruit ++	11 (5 Ma; 6 Mi)	Af, Hg

Table S1 Continued

Plant species and voucher number	Plant family	Local names	Local food category	Part used and extent of use	Cited by % of respondents	Habitat
Artocarpus elasticus Reinw. ex Blume (LP21)	Moraceae	Tarok (Mi); Torop (Ma)	Fruit	Fruit<br>+	10 (3 Ma; 7 Mi)	Af, Fo
Syzygium malaccense (L.) Merr. & L.M.Perry	Myrtaceae	Jambu bol (Mi, Ma); Jambak (Mi)	Fruit	Fruit<br>++	8 (3 Ma; 5 Mi)	Af, Fo, Hg
Ficus sp.	Moraceae	Limpauang (Mi); Gala-gala (Ma)	Fruit	Fruit<br>-	7 (3 Ma; 4 Mi)	Af, Fo
Ananas comosus (L.) Merr.	Bromeliaceae	Nanas, Naneh (Mi); Onas, Nenas (Ma)	Fruit	Fruit<br>++	5 (3 Ma; 2 Mi)	Af, Fi, Hg
Lansium parasiticum (Osbeck) K.C.Sahni & Bennet	Meliaceae	Aia-aia, Aie-aie (Mi)	Fruit	Fruit<br>+	4 (0 Ma; 4 Mi)	Af, Hg
Physalis angulata var. *Angulata* (LP62)	Solanaceae	Lapuik-lapuik , Pultak-pultak (Ma); Ciplokan (Mi)	Fruit	Fruit<br>++	4 (3 Ma; 1 Mi)	Af, Fi
Nephelium mutabile Blume (LP46)	Sapindaceae	Polesan (Mi)	Fruit	Fruit<br>+	4 (0 Ma; 4 Mi)	Af, Hg
Solanum americanum Mill. (LP19)	Solanaceae	Buah lumai (Mi); Buah ranti (Ma)	Fruit	Fruit<br>-	2 (0 Ma; 2 Mi)	Af, Fi, Hg
Lansium parasiticum (Osbeck) K.C.Sahni & Bennet	Meliaceae	Garandan, Barandan (Mi)	Fruit	Fruit<br>+	2 (0 Ma; 2 Mi)	Af, Hg
Callicarpa arborea Roxb.	Lamiaceae	Sinasi, Nasi-nasi (Mi)	Fruit	Fruit<br>-	0 (0 Ma; 1 Mi)	Ag, Fi
**Food group: Vitamin A-rich plants**						
Carica papaya L.	Caricaceae	Pepaya (Mi); Botiak liar (Ma)	Fruit	Fruit<br>++++	45 (22 Ma; 23 Mi)	Af, Fi, Hg
Passiflora foetida L. (LP29)	Passifloraceae	Latuik-latuik, Markisa hutan (Mi, Ma); Rambutan akar (Mi); Sigambus (Ma)	Fruit	Fruit<br>+++	44 (38 Ma; 44 Mi)	Af, Fi, Fo
Mangifera foetida Lour.	Anacardiaceae	Ambacang, Bacang, Macang, Mangga hutan (Mi); Ambacam (Ma)	Fruit	Fruit<br>++	30 (10 Ma; 20 Mi)	Af, Fo
Mangifera laurina Blume (LP05)	Anacardiaceae	Polam, Ampalam (Mi); Manga harrangan, Manga kampung (Ma)	Fruit	Fruit<br>+	15 (2 Ma; 13 Mi)	Af, Fo
Mangifera quadrifida Jack (LP52)	Anacardiaceae	Languang, Polam, Mangga hutan (Mi)	Fruit	Fruit<br>+	13 (0 Ma; 13 Mi)	Af, Fo

Abbreviations: Ae – aquatic environments, Af – agroforestry, Fi - fields, Fo – forests, Hg – home gardens, Rf – rice fields. Mi – Minangkabau language and ethnic, Ma – Mandailing language and ethnic. The extent of use is based on 4-cell analysis where: - means only past use; + used rarely by a few households; ++ used rarely by many households; +++ used frequently by a few households; ++++ used frequently by many households
